# Coding complete sequences of the 10 genomic segments of a mammalian orthoreovirus type 3 isolated from a *Blarina peninsulae* shrew

**DOI:** 10.1128/mra.00219-25

**Published:** 2025-06-10

**Authors:** Emily DeRuyter, Robert A. Williams, Kuttichantran Subramaniam, John A. Lednicky

**Affiliations:** 1 Department of Environmental and Global Health, College of Public Health and Health Professions, University of Florida 271714, Gainesville, Florida, USA; 2 Emerging Pathogens Institute, University of Florida 145775, Gainesville, Florida, USA; 3 Department of Infectious Diseases and Pathology, University of Florida College of Veterinary Medicine 70122, Gainesville, Florida, USA; Katholieke Universiteit Leuven, Leuven, Belgium

**Keywords:** virus discovery

## Abstract

Mammalian orthoreoviruses affect a wide array of mammals including humans. Here, we present the draft genome assembly of a mammalian orthoreovirus isolated from a *Blarina peninsulae* shrew.

## ANNOUNCEMENT

Mammalian orthoreovirus is a species within the genus *Orthoreovirus* of the family *Spinareoviridae*, formerly *Reoviridae* (1). The genome consists of 10 linear dsRNA segments. There are four mammalian orthoreovirus serotypes: type 1 Lang, type 2 Jones, type 3 Dearing, and type 4 Ndelle, collectively referred to as mammalian reoviruses (MRV) (1). Antibodies used to define MRV serotypes target the σ1 protein, which is responsible for viral attachment on host cell receptors (2). There is extensive sequence identity between the proteins encoded by homologous MRV genome segments (1). Mammalian reoviruses have been detected in humans, white-tailed deer, bats, and other mammals, where infections can result in respiratory, central nervous system, and enteric diseases (3–12).

Samples of spleen and kidney tissues were obtained from a dead *Blarina peninsulae* shrew in 2024 that had been caught and killed by a pet cat in Gainesville, Florida. Aliquots of the tissue samples were processed as previously described (13) and inoculated onto nearly confluent monolayers of Tb1Lu (*Tadarida brasiliensis* [free-tailed bat] lung, CCL #88) and Vero E6 cells (*Cercopithecus aethiops* [African green monkey]) obtained from the American Type Culture Collection (ATCC, Manassas, VA, Cat#: ATCC CRL1586). Virus-induced cytopathic effects (CPEs) in Vero E6 cells were first observed 7 days post-inoculation (dpi). The CPE consisted of cytoplasmic inclusions and granulation of the cells followed by the detachment of dead cells from the growing surface.

Virus RNA was extracted from virions in the spent media of Vero E6 cells 10 dpi using a QIAamp Viral RNA Mini Kit (Qiagen, Germany). A cDNA library was generated using a NEBNext Ultra RNA Library Prep Kit (New England Biolabs) and sequenced using an Illumina NextSeq1000 sequencer (Illumina, Inc., San Diego, CA). The sequencing run generated a total of 9,000,338 reads with an average read length of 261 bp. *De novo* assembly of the untrimmed paired-end reads was performed using MEGAHIT v1.1.4 with default parameters (14). A BLASTX search of the resulting contigs was performed using OmicsBox, identifying all 10 coding-complete genomic segment sequences of a mammalian orthoreovirus. Additionally, mapping the raw reads back to these sequences in CLC Genomics Workbench v20.0.4 revealed an average coverage of 2,119 reads per nucleotide. Salient features of the genomic sequence are presented in Table 1.

**TABLE 1:** Inferred lengths of structural and non-structural proteins of Gainesville shrew mammalian orthoreovirus type 3 strain UF-1

Segment	Segment length^*a*^	Class	AA size	Protein function	Similar genes	Accession number
L1	3,831 bp	λ3	1,267	RNA-dependent RNA polymerase	Mammalian orthoreovirus isolate T1E1v7	OR074618.1
L2	3,901 bp	λ2	1,289	Capping enzyme with guanylyl and methyl transferase activity; turret protein	Mammalian orthoreovirus isolate 17-EF40	MW718863.1
L3	3,882 bp	λ1	1,275	Binds dsRNA, helicase, NTPase, and RNA triphosphatase	Mammalian orthoreovirus 2 isolate OV204	MK092964.1
M1	2,168 bp	μ2	736	NTPase; inclusion body development; cell tropism; modulation of cellular interferon response	Mammalian orthoreovirus strain MRV2 809/Bat/Nebraska USA/2017	OP057395.1
M2	2,188 bp	μ1; μ1C	708	µ1 is cleaved to µ1C and myristolyated µ1N after assembly; during viral entry (cell penetration), µ1C is further cleaved to δ and φ; µ1C multimerizes with σ3 in a *T* = 13 symmetry in the outer capsid; involved with transcriptase activation	Mammalian orthoreovirus 2 Osaka2014	LC476918.1
M3	2,223 bp	μNS; μNSC	721	Viral factory protein. Binds ssRNA and cytoskeleton; nucleates viral inclusion bodies; µNSC (unknown function) derived from alternate translation start site	Mammalian orthoreovirus isolate 17-EF40	MW718867.1
S1	1,385 bp	σ1; σ1s	455	σ1 = viral attachment protein; homotrimer; cell tropism; pathways of viral spread *in vivo*; σ1s = viral spread *in vivo*; cell cycle arrest	Mammalian orthoreovirus 3	NC_013231.1
S2	1,316 bp	σ2	418	Binds dsRNA; inner capsid structural protein assumes a *T* = 1 symmetry	Mammalian orthoreovirus 2 isolate OV204	MK092971.1
S3	1,152 bp	σNS	366	Inclusion body formation, binds ssRNA; RNA stability	Mammalian orthoreovirus isolate T2E2	OR074537.1
S4	1,169 bp	σ3	365	Binds dsRNA; multimerizes with µ1; nuclear and cytoplasmic localization; translation control; modulation of cellular interferon response	Mammalian orthoreovirus isolate T2E3	OR074548.1

^*a*^ Nearly complete segment sequence; contains coding complete sequence.

**Fig 1:**
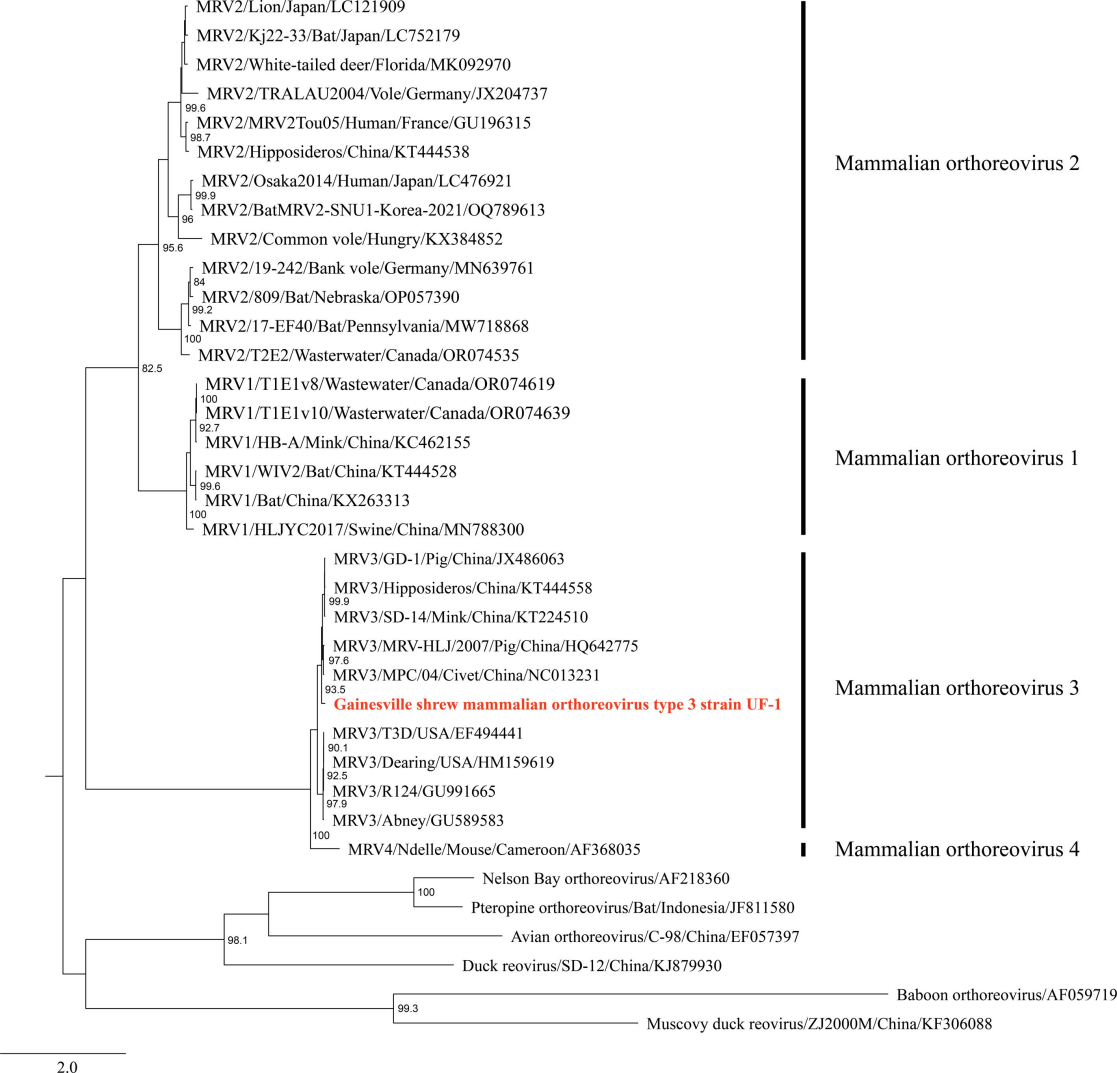
ML phylogenetic tree of Gainesville shrew mammalian orthoreovirus type 3 strain UF-1.

A maximum likelihood (ML) analysis based on nucleotide sequence alignment of the outer capsid protein (*σ1*) gene was performed. The resulting ML tree supported this virus as a strain of mammalian orthoreovirus type 3 (Fig. 1, with a 92.62% sequence identity with mammalian orthoreovirus 3 segment S1 [NC_013231.1]). We designated the virus as Gainesville shrew mammalian orthoreovirus type 3 strain UF-1.

Maximum likelihood phylogram depicting the relationship between the Gainesville shrew mammalian orthoreovirus type 3 strain UF-1 (highlighted in red) and 35 other orthoreoviruses, based on the nucleotide sequence alignment of the outer capsid protein (σ1) gene performed using MEGA 11 with 1,000 bootstraps model GTR + G (15). Bootstrap support values ≥80% are indicated at nodes.

## Data Availability

The 10 coding-complete genome sequences are available in GenBank (accession numbers PQ869305.1 to PQ869314.1). Raw reads are available under https://www.ncbi.nlm.nih.gov/sra/SRR31992870.
